# Rectal cancer level significantly affects rates and patterns of distant metastases among rectal cancer patients post curative-intent surgery without neoadjuvant therapy

**DOI:** 10.1186/1477-7819-12-197

**Published:** 2014-06-30

**Authors:** Jy Ming Chiang, Pao Shiu Hsieh, Jinn Shiun Chen, Reiping Tang, Jeng Fu You, Chien Yuh Yeh

**Affiliations:** 1 Division of colorectal surgery, Department of surgery, Chang Gung Memorial Hospital, Lin-Kou medical center, and College of Medicine, Chang Gung University, No.5, Fu-Hsing St. Kuei-Shan, Tao-Yuan 333, Taiwan; 2 Chang Gung University, College of Medicine, Tao-yuan 333, Taiwan

## Abstract

**Background:**

Rectal cancer patients have a higher incidence of pulmonary metastases than those with colon cancer. This study aimed to examine the effects of rectal cancer level on recurrence patterns in rectal cancer patients.

**Methods:**

Patients with T3/T4 rectal cancers who underwent surgery between 2002 and 2006 were recruited in this study. All the patients were followed up on until death. Recurrence patterns and survival rates were calculated in relation to clinical variables.

**Results:**

There were 884 patients were enrolled in this study. Patients with low-rectal cancer had significantly worse five-year overall survival (OS) and disease-free survival (DFS) rates (47.25% and 44.07%, respectively) than patients with mid-rectal (63.46% and 60.22%, respectively) and upper-rectal cancers (73.91% and 71.87%, respectively). The level of the tumor (*P* <0.001), nodal status (*P* <0.001), tumor invasion depth (*P* <0.001), and tumor differentiation (*P* = 0.047, *P* = 0.015) significantly affected the surgical outcomes related to OS and DFS in the univariate and multivariate analyses. Furthermore, the level of the rectal cancer was a significant risk factor (hazard ratio 1.114; 95% CI, 1.074 to 1.161; *P* <0.001) for local recurrence, lung metastases, bone metastases, and systemic lymph node metastases. Significantly higher incidence rates of bone (53.8%) and brain metastases (22.6%) after initial lung metastases rather than initial liver metastases (14.8% and 2.9%, respectively) were also observed.

**Conclusions:**

For rectal cancer patients who underwent surgical resection, the rectal cancer level significantly affected surgical outcomes including rates and patterns of distant metastases.

## Background

Over the past two to three decades, total mesorectal excision [1] and neoadjuvant chemoradiation therapy have been widely adopted to reduce local recurrence [2] and improve the surgical outcome of rectal cancer. Unfortunately, most studies have not been able to show any significant improvement in survival because the frequency of distant metastases remained high [2-5]. Moreover, patients with distal rectal cancer who underwent abdominoperineal resection (APR) had worse oncological outcomes than those who underwent anterior resection [6]. These data raised the possibilities that low-rectal cancer possesses a distinct biological behavior or that extended APR might require a more radical excision [7,8].

Previous reports have shown that rectal cancer patients have a much higher incidence of synchronous and metachronous pulmonary metastases than those with colon cancer [9-11]. Tan *et al.*[9] reported a 12% versus 6% rate of synchronous lung metastases for primary rectal cancer compared with primary colon cancer. Similarly, Mitry *et al.*[10] demonstrated that compared with colon cancer, rectal cancer showed a 2.8-fold increase (16.7% versus 7.5%, respectively) in the five-year cumulative rate of synchronous lung metastases and a 2.63-fold increase (9.1% versus 4.1%, respectively) in the rate of metachronous lung metastases. Some clinicopathological factors such as nodal status and tumor invasion depth were also found to significantly affect surgical outcomes in these studies [9-12]. However, the impact of the rectal cancer level on local recurrences or distant metastases remains unknown.

Here, we retrospectively analyzed the surgical outcomes of rectal cancer patients who did not undergo neoadjuvant chemoradiation therapy to characterize the rates and patterns of recurrence in relation to locations of rectal cancer and clinicopathological factors.

## Methods

### Registry and patients

The database of the Colorectal Cancer Registry of the colorectal surgery section in Chang Gung Memorial Hospital (CGMH) was retrospectively reviewed for this study*.* This registry was first established in 1985 and a revised data record form was implemented in 1995. The data collected included a detailed family history, demographic variables, preoperative evaluation, operation records, and postoperative follow-ups. The extent of the cancer at the time of diagnosis was classified according to the American Joint Committee on Cancer TNM staging system and Astler-Coller modified Dukes’ staging system after surgery, with a review of the pathologic specimen and investigation of distant metastases. Tumor locations were classified according to the International Classification of Diseases. The status at follow-up was updated every year for the surviving patients. All the data were recorded by surgical nursing specialists on a standardized form through patient interviews and from clinical and pathological records. All the data were confirmed by one of the authors (RP Tang or DF You) before being translated into a numeric code and keyed into the computer by a registry staff member (SN Lin). Follow-up data were added annually by reviewing patients’ records on medical charts. A telephone interview or mailed questionnaire was used if a patient’s medical records were not available. The study was approved by the Institutional Review Board of CGMH.

The records of all the patients with rectal cancers who underwent surgery between 1 January 2002, and 31 December 2006 were retrieved. Demographic and clinical variables were recorded including sex, age, tumor histological grade, tumor location, tumor level (distances from the anal verge) for rectal cancer, lymph node status, operation morbidity and mortality, and follow-up status. The tumors were graded on the basis of the World Health Organization classification and were staged by TNM classification.

Distal, middle, and upper-rectal cancers were defined as tumors located less than 5.1, 5.1 to 10, and 10.1 cm or more above the anal verge. Preoperative staging was performed by physical examination, rigid sigmoidoscopy, and colonofiberscopy. Endorectal ultrasound was sometimes required for tumors closer than 10 cm from the anal verge. Routine chest to abdominal computerized tomography scans (CT) were performed to survey distant metastases.

### Follow-up

After colorectal tumor resection, the patients were enrolled in a follow-up program that involved outpatient visits every three to four months for the first two postoperative years, with physical examination and carcinoembryonic antigen tests. A chest X-ray, abdominal sonography or computer-assisted tomography scan, and colonoscopy were performed every one to three years after the operation and whenever necessary. All the patients were followed up on until death or January 2012, whichever came first. Recurrence was histologically confirmed by biopsy, reoperation, imaging study, elevation of tumor marker, or a combination of these. The index date for the determination of survival time was defined as the date of colorectal cancer surgery. Disease-free survival time was the interval between surgery and the date of recurrence. Overall survival was calculated between date of surgery and last department contact (scheduled follow-up, mailed response, or telephone contact) or patient death (verified by medical record or death certificate).

### Statistical analyses

The data were entered into Excel 2000 (Microsoft Corporation, Redmond, Washington, United States) and analyzed using the Statistical Package for the Social Sciences (release 13.0; SPSS Inc., Chicago, Illinois, United States). The chi-squared test for trends or the Fisher exact test was used whenever appropriate. Overall survival (OS) and disease-free survival (DFS) rates were calculated using the Kaplan-Meier method, and the log-rank test was used to compare the significance of the differences between the groups. Cox proportional hazards regression was used to assess the individual contribution of the factors associated with recurrences. All the variables that were significant in the univariate analysis were entered into a multivariate analysis. All the tests were two-sided and all the results are presented with their hazard ratios (HR) and 95% confidence intervals (CI). Statistical significance was set at *P* <0.05.

## Results

### Patients and clinicopathological features

From 1 January 2002 to 31 December 2006, 1810 patients were found with rectal adenocarcinoma and underwent resection at the CGMH, including 58 Tis, 144 T1, 292 T2, 766 T3, 516 T4, and 34 T staging-unknown patients. From the 1282 patients with T3 or T4 rectal cancers, 279 had synchronous distant metastases. Of the remaining 1003 T3/T4 patients without synchronous distant metastases, 119 accepted preoperative neoadjuvant therapy, either short-course radiation therapy (total dose, 2500 cGy at 500 cGy 5 times) or long-course chemoradiation therapy (CCRT; total dose 5040 cGy at 180 cGy 28 times, combined with 5-Fu chemotherapy) based on the surgeon’s advice. These patients were excluded and the remaining 884 patients were enrolled in this study. Surgical resection was performed according to the principle of total mesorectal excision. The mean follow-up period was 77.8 months (range: 20.0 to 154 months, standard deviation: 32.7 months).

The clinicopathological characteristics of these patients according to the level of the rectal cancer (distal rectum, 0 to 5 cm above the anal verge; mid-rectum, 5.1 to 10 cm above the anal verge; and upper rectum, 10.1 to 15 cm above the anal verge) are summarized in Table 1. No significant differences were observed in these features in terms of rectal tumor level.

**Table 1:** Clinicopathological features of patients with rectal adenocarcinoma involving different locations along the rectum

**Location of the tumor above the anal verge (cm)**	**0-5 cm**	**5.1-10 cm**	**10.1-15 cm**
Total number of patients with tumors at different locations	117	331	436
Number of female patients (%)	61 (52.14%)	151 (45.62%)	192 (44.04%)
Number of male patients (%)	56 (47.86%)	180 (54.38%)	244 (55.96%)
Age, mean ± SD (range) years	67 ± 14 (30-96)	64 ± 14 (30-90)	65 ± 13 (24-95)
Degree of differentiation			
Well	11 (9.40%)	32 (9.67%)	42 (9.63%)
Moderate	96 (82.05%)	285 (86.10%)	375 (86.01%)
Poor	10 (8.55%)	14 (4.23%)	19 (4.36%)
Tumor invasion depth			
T3	83 (70.94%)	221 (66.67%)	259 (59.40%)
T4	34 (29.06%)	110 (33.23%)	177 (40.60%)
Lymph node status			
N0	53 (45.30%)	147 (44.41%)	194 (44.50%)
N1	27 (23.08%)	84 (25.38%)	138 (31.65%)
N2	37 (31.62%)	100 (30.21%)	104 (23.85%)

### Treatment outcomes

Surgical outcomes including OS and DFS were shown to be related to the level of the rectal cancer (Table 2). Significantly worse one, three, and five-year OS and DFS rates were observed for the distal rectum (88.87, 59.33, and 47.25%, and 66.93, 47.39, and 44.07%, respectively) and mid-rectum (94.86, 78.21, and 63.46%, and 84.04, 67.31, and 60.22%, respectively) than for the upper rectum (94.46, 81.41, and 73.91%, and 86.83, 75.11, and 71.87%, respectively).

**Table 2:** Overall survival and disease-free survival of patients with rectal adenocarcinoma involving different locations along the rectum

**Tumor location**	**Total patient no.**	**Overall survival**						**Disease-free survival**					
		**1 year (%)**	**3 years (%)**	**5 years (%)**	**HR**	**95.0% CI**	***P***	**1 year (%)**	**3 years (%)**	**5 years (%)**	**HR**	**95.0% CI**	***P***
10.1-15 cm	436	94.46	81.41	73.91	1			86.83	75.11	71.87	1		
5.1-10 cm	331	94.86	78.21	63.46	1.263	1.004-1.589	0.046	84.04	67.31	60.22	1.508	1.177-1.932	0.001
0-5 cm	117	88.87	59.33	47.25	2.092	1.571-2.786	<0.001	66.93	47.39	44.07	2.548	1.876-3.46	<0.001

The results of the univariate analyses of the clinicopathological features as prognostic factors for OS and DFS were performed and are shown in Table 3. The results of the multivariate analysis for significant prognostic factors from the univariate analyses are shown in Table 4. Nodal status (*P* <0.001), tumor invasion depth (*P* <0.001), tumor differentiation (*P* = 0.047, *P* = 0.015), and rectal cancer level (*P* <0.001) were significant factors affecting OS and DFS in the univariate and multivariate analyses after adjusting for age and sex.

**Table 3:** Univariate analysis of the clinicopathological features as prognostic factors for overall survival and disease-free survival

	**Overall survival**			**Disease-free survival**		
**Variables**	**HR**	**95.0% CI for HR**	***P***	**HR**	**95.0% CI for HR**	***P***
Age	1.024	1.015-1.033	<0.001	0.995	0.987-1.004	0.305
Sex						
Female	1	-	-	1	-	-
Male	1.237	1.003-1.525	0.047	0.956	0.766-1.193	0.689
Location along the rectum						
10.1-15 cm	1			1		
5.1-10 cm	1.263	1.004-1.589	0.046	1.508	1.177-1.932	0.001
0-5 cm	2.092	1.571-2.786	<0.001	2.548	1.876-3.460	<0.001
TMN_T						
T3	1	-	-	1	-	-
T4	1.436	1.166-1.770	0.001	1.606	1.285-2.006	<0.001
TMN_N						
N0	1	-	-	1	-	-
N1	1.543	1.186-2.008	0.001	2.155	1.599-2.904	<0.001
N2-N3	2.435	1.904-3.114	<0.001	3.751	2.847-4.941	<0.001
HG						
Well	1	-	-	1	-	-
Mod	1.039	0.732-1.475	0.830	1.313	0.871-1.979	0.193
Poor	2.194	1.313-3.669	0.003	2.879	1.644-5.043	<0.001

**Table 4:** Multivariate analysis of significant prognostic factors identified by univariate analysis

		**Overall survival**			**Disease-free survival**		
**Variables**		**HR**	**95.0% CI for HR**	***P***	**HR**	**95.0% CI for HR**	***P***
Age		1.026	1.017-1.035	<0.001	-	-	-
Sex							
Female	1	-	-	-	-		-
Male	1.260	1.019-1.558	0.033	-	-		-
Location along the rectum							
10.1-15 cm	1				1		
5.1-10 cm	1.315	1.044-1.657	0.020	1.570	1.224-2.014		<0.001
0-5 cm	2.284	1.69-3.03	<0.001-	2.780	2.038-3.792		<0.001
TMN_T							
T3	1	-	-	1	-		-
T4	1.480	1.195-1.835	<0.001	1.593	1.268-2.001		<0.001
TMN_N							
N0	1	-	-	1	-		-
N1	1.693	1.296-2.211	<0.001	2.194	1.623-2.966		<0.001
N2-N3	2.454	1.902-3.166	<0.001	3.377	2.543-4.485		<0.001
HG							
Well	1	-	-	1	-		-
Mod	0.891	0.622-1.287	0.530	1.056	0.695-1.604		0.798
Poor	1.710	1.008-2.899	0.047	2.044	1.152-3.627	0.015	

### Risk of recurrences related to location of rectal cancers

The risk of local recurrence or different distant metastases according to the location of the rectal cancer was further analyzed and the results are summarized in Table 5. The site shown in this table indicated as the site of recurrence refers to either local recurrence or distant metastases detected during the follow-up period. In this study, isolated recurrence occurred in 167 patients (44%) and multiple recurrences (present in two or more organs at the same time during follow-up) were discovered among 209 patients (55%). The location of the rectal cancer was found to be a significant risk factor for local recurrence, lung metastases, bone metastases, and systemic lymph node metastases. For example, lung metastasis was higher in distal rectal cancer and mid-rectal cancer than in upper-rectal cancer (mid-rectal cancer: HR, 1.760; *P* = 0.001; and low-rectal cancer: HR, 3.187; *P* <0.001). In other words, the risk of lung metastases was more than three times higher for the distal rectum than for the upper rectum (Table 5). In general, as the distance of the rectal cancer relative to the anal verge decreased, the HR for recurrences significantly increased (HR, 1.114; 95% CI, 1.074 to 1.161; *P* <0.001 for each 1 cm difference).

**Table 5:** Correlation of the risk of local recurrence and patterns of distant metastases with the location of the adenocarcinoma along the rectum

**Adenocarcinoma location**	**10.1-15 cm (n = 436)**				**5.1-10 cm (n = 331)**					**0-5 cm (n = 117)**				
	**Patient no. (%)**		**HR**	**95% CI**	**Patient no. (%)**		**HR**	**95% CI**	***P***	**Patient no. (%)**		**HR**	**95% CI**	***P***
**Recurrence in patients/Recurrence patterns**														
Local	36	(8.26)	1	-	50	(15.11)	1.885	1.23-2.89	0.004	23	(19.66)	2.786	1.65-4.70	<0.001
Lung	57	(13.07)	1	-	73	(22.05)	1.760	1.25-2.49	0.001	38	(32.48)	3.187	2.11-4.81	<0.001
Liver	58	(13.3)	1	-	45	(13.06)	1.012	0.69-1.49	0.951	12	(10.26)	0.832	0.45-1.55	0.563
Bone	15	(3.44)	1	-	23	(6.95)	2.090	1.09-4.01	0.026	13	(11.11)	4.043	1.92-8.51	<0.001
Brain	6	(1.38)	1	-	8	(2.42)	1.819	0.63-5.24	0.268	4	(3.42)	3.136	0.88-11.13	0.077
Carcinomatosis	8	(1.83)	1	-	9	(2.72)	1.494	0.58-3.87	0.409	5	(4.27)	2.649	0.87-8.10	0.088
Systemic LN	15	(3.44)	1	-	31	(9.37)	2.788	1.51-5.17	0.001	17	(14.53)	5.046	2.52-10.11	<0.001

### Sequential metastases after local recurrence, lung, and liver metastases

Since local, liver, and lung were the three most common sites of recurrences, the rates and patterns of sequential metastases after recurrence at these sites or organs are summarized and compared in Table 6. During the follow-up period isolated metastases were found among 140 patients, including 53 lung metastases, 42 liver metastases, and isolated local recurrence in 45 patients. Multiple organ metastases were found in 176 patients; sequential metastases after initial local recurrence, initial lung, or initial liver metastases are shown in Table 6. Significantly higher rates of bone (53.8%) and brain metastases (22.6%) were observed after initial lung metastases than after initial liver metastases (14.7% and 2.9%, respectively) and initial local recurrence (10.7% and 3.6%, respectively). In addition, higher lung metastases (78.6%) were found after initial liver recurrence. A significant difference in the mean time that brain metastases were detected after liver (732 days), lung (345 days), and local recurrence (398 days, *P* <0.01) were also observed. Furthermore, brain metastases were found to be significantly higher (22.6% versus 3.6% and 2.9%, respectively) after lung metastases. It is interesting that liver metastases (15.4%) were also detected after lung metastases, with a mean delayed time of 272 days. Local recurrences were detected after liver and lung metastases (11.5% and 10.7%, respectively).

**Table 6:** Sequential metastases after lung and liver metastases

	**Local first**			**Lung first**			**Liver first**			
	**Number of patients (%)**			**Number of patients (%)**			**Number of patients (%)**			***P***
	**Solitary**	45		**Solitary**	53		**Solitary**	42		
	**Multiple**	68	(100)	**Multiple**	52	(100)	**Multiple**	56	(100)	
**Multiple Organs**	**Lung**	32	(47.0)	**Local**	6	(11.5)	**Local**	6	(10.7)	
	**Liver**	23	(33.9)	**Liver**	8	(15.4)	**Lung**	44	(78.6)	
	**Bone**	10	(14.7)	**Bone**	28	(53.8)	**Bone**	6	(10.7)	0.001
	**Brain**	2	(2.9)	**Brain**	12	(22.6)	**Brain**	2	(3.6)	0.010
	**Carcinomatosis**	7	(10.3)	**Carcinomatosis**	6	(11.5)	**Carcinomatosis**	3	(5.4)	0.185
	**Systemic LN**	22	(32.3)	**Systemic LN**	16	(30.7)	**Systemic LN**	18	(32.1)	0.120

## Discussion

Here, we have demonstrated that in addition to nodal status, tumor invasion depth, and tumor differentiation, location of the rectal cancer also significantly affects the surgical outcome (OS and DFS) of patients who did not undergo neoadjuvant chemoradiation therapy. Compared with those with upper-rectal cancer, patients with distal and mid-rectal cancers were more likely to have distant metastases, including lung, bone, and systemic lymph node metastases. A significant increase in the frequency of developing lung metastases was observed for distal and mid-rectal cancers (3.18-fold and 1.76-fold, respectively). Similarly, significant increases in the frequencies of bone metastases (4.04-fold and 2.09-fold for distal and mid-rectum, respectively) and systemic lymph node metastases (5.04-fold and 2.78-fold for distal and mid-rectum, respectively) were also observed. To our knowledge, this is the first report revealing the influence of rectal cancer location on distant metastases patterns among patients without CCRT, and the findings presented here may impact several aspects of clinical practice.

First, our data may influence the preoperative staging protocol and postoperative follow-up strategy. Some studies question the clinical value of routine preoperative chest CT’s and indicate that it does not influence therapeutic strategy in colorectal cancer patients. This is because interpretation of the nature of pulmonary lesions is not always easy and only a small proportion of these lesions are actually malignant [13]. However, other studies have shown that a chest CT should be recommended as a routine staging method at least for low- and mid-rectal cancers because of their higher rates of lung metastases [9-11]. In addition, a preoperative chest CT is useful for the early detection of pulmonary metastases as a baseline study for abnormal lung nodules [12]. Currently, a wide variety of imaging methods are available for colorectal cancer staging, thus, selection based on the particular organs involved and high-risk sites may be important.

Second, our data might provide a baseline reference for comparisons of treatment outcomes with and without neoadjuvant chemoradiation therapy. Many previous neoadjuvant CCRT studies have shown that although reduced local recurrence could be achieved, survival was not significantly improved because the frequencies of distant metastases remained high [2-5]. Furthermore, a recent report showed that pulmonary recurrence predominates after combined chemoradiation therapy and surgical resection for locally advanced rectal cancer patients [14]. The study demonstrated that post-resection rectal cancer without neoadjuvant chemoradiation showed a higher risk of lung metastases for lower-lying rectal cancers, similar to the findings presented here. Thus, the ratio of different levels of rectal cancer patients included in different studies may significantly affect the observed recurrence patterns and rates in the same way that T or N stages do. Based on these results, the prevention of systemic metastases should be emphasized during the introduction of more effective neoadjuvant chemotherapy. This is especially important for subgroups of patients with one or more risk factors such as low- and mid-rectal cancers, high degrees of invasion of the bowel wall, and involvement of local lymph nodes.

The significant increase of distant metastases for distal and mid-rectal cancers may be multifactorial rather than related to an increase in local recurrence, as only 17.7% (32 of 181 patients) of lung metastases were followed by local recurrence with a mean time of 271 days (Table 6). These increased lung and other distant metastases may be explained by existing anatomic reasons. Anatomically, the rectum is approximately 15 cm in length commencing from the sacral promontory to the anorectal ring. The rectum is usually described as having three segments: an upper, a middle, and a lower. Lymphatic drainage accompanied with blood supplies to these three segments are from the superior, middle, and inferior rectal artery and vein. The superior rectal vein is drained into the inferior mesenteric vein and then to the portal system, while the inferior rectal vein drains into the common iliac vein and to the inferior vena cava, and middle rectum drainage may be both cephalad to the inferior vein and lateral to the internal iliac vein. Therefore a distal rectal tumor may easily metastasize initially to the lungs because the inferior rectal vein drains into the inferior vena cava, bypassing the portal venous system. Basically, distant metastasis of cancer is presumed to occur via lymphatic or vascular drainage routes, and the liver is the first organ of the reticuloendothelial system that would allow for the initial dissemination of colon and upper-rectal cancers entering through the portal vein. Thus, the liver is clinically reported to be the most common site of distant metastases. However, if the drainage of distal or mid-rectal cancer cells were via the inferior and middle rectal vein into the inferior vena cava (systemic circulation) rather than drainage into the portal system (such as the colon and upper rectum via the inferior mesenteric vein), then the metastasis patterns might differ based on the location of the rectal cancer. Unfortunately, very few papers have described the existing anatomic factors that significantly affect metastasis variances; for example, for lower rectal cancer, systemic venous circulation plays a more important role in the metastatic process.

Distant metastases basically follow mechanical vascular spreading, a ‘cascade’ process of metastasis of metastases described by Viadana *et al.*[15]. This process might be also affected by a process described by the ‘seed and soil’ theory [16], which indicates that delivery of cancer cells to different organs varies in efficiency [16,17]. However, the seed and soil theory is not mutually exclusive with hemodynamic theories [17]. In this study, our data highlights the importance of vascular parameters related to the metastatic pattern. Most cases (156 of 176, 88.6%) followed the theory of mechanical vascular spreading (Table 6). However, the findings that 11.4% (20 of 176 cases; 6 lung metastases followed by local recurrence, 8 lung metastases followed by liver metastases, and 6 liver metastases followed by local recurrence; Table 6) may indicate variable efficiencies in cancer cell delivery to different organs. This result may point to the fact that site and growth rate in different organs may be two independent processes for metastases. In summary, our study emphasizes that mechanical vascular-spreading theories predominantly determine the disseminating site of metastases, whereas the rate of metastasis growth may also be controlled by other factors such as the seed and soil theory.

This study provides important insight into the effects of rectal cancer location on recurrence patterns; however, this study had some limitations. First, we did not have data on the distance of the circumferential resection margin in our database for this study period (2002 to 2006). Our data registry was revised in 2009 to include circumferential resection margin status. This is recorded in the histology report of the tumor specimen, which is prepared by a pathologist. However, the high local recurrence rate for low-lying tumors may be related to the high rates of a positive circumferential resection margin. Second, during the study period, we did not adopt the cylindrical excision technique by Holm [18]. We may well consider that this technique will improve surgical outcomes for low-lying rectal cancer in terms of local recurrence. However, to what extent this technique contributes to distant metastases rates, such as the high rate of lung metastases, remains unclear. Third, although T3/T4 rectal cancer patients with or without lymph node involvement were included in this retrospective study, preoperative neoadjuvant chemoradiation therapy was not routinely applied, as this was not adopted as a guideline in our hospital until 2007. During the years 2002 to 2006, 119 patients who accepted preoperative neoadjuvant radiation therapy or CCRT based on the individual surgeon’s preference were excluded from this study. Fourth, not all diagnosed distant metastases in this series were diagnosed through confirmed histological or cytological examination results, thus some patients may not have been differentially evaluated from those who had other secondary primaries or just post-infective scarring.

## Conclusions

In summary, the level of the rectal cancer significantly affected surgical outcomes, including rates and patterns of distant metastases for the patients who underwent surgical resection. Lung, bone, and systemic lymph node metastases significantly increased as the level of the rectal cancer decreased.

## Competing interests

The authors declare that they have no competing interests.

## Authors’ contributions

Concept and design: JMC, CYY. Provision of study patients: PSH, CYY, and JMC. Collection and assembly of data: PSH, JSC, JFY, and JMC. Data analysis and interpretation: JMC, CYY, JFY. Manuscript writing: JMC, RT. Final approval of manuscript: PSH, CYY, JSC, and JMC, JFY, RT. All authors read and approved the final manuscript.
